# 

**Published:** 1871-02

**Authors:** 


VOL. I. FEBRUARY, 1871. NO. 2.

### THE GEORGIA

### Medical Companion,

*A MONTHLY ADVISER,*

DEVOTED TO THE

SCIENCE OF MEDICINE AND SURGERY.

EDITED BY

T. S. POWELL, M. D.,
W. T. GOLDSMITH, M. D.

*"QUICQUID PRÆCIPIES ESTO BREVIS."*

### CONTENTS.

PART I.—ORIGINAL COMMUNICATIONS AND SPECIAL SELECTIONS.
PART II.—ABRIDGED EXTRACTS AND GLEANINGS FROM OUR EXCHANGES.
PART III.—BIOGRAPHICAL, ETHICAL, HYGENIC, AND MISCELLANEOUS.
PART IV.—EDITORIALS, REVIEWS, ITEMS, AND NEWS.

ATLANTA, GA.
FRANKLIN STEAM PRINTING HOUSE.
J. J. TOON, PROPRIETOR.

TERMS, - - - - - - $2 A YEAR IN ADVANCE.

